# Effect of angiotensin converting enzyme inhibitors and angiotensin receptor blockers on hemoglobin levels

**DOI:** 10.1186/1756-0500-6-443

**Published:** 2013-11-04

**Authors:** Adnan Ajmal, Charles E Gessert, Brian P Johnson, Colleen M Renier, Jeanette A Palcher

**Affiliations:** 1 Hospital Medicine, UMass Memorial HealthAlliance Hospital, Leominster, MA, USA; 2 Essentia Institute of Rural Health, Duluth, MN, USA

**Keywords:** Angiotensin converting enzyme inhibitors (ACEIs), Angiotensin receptor blockers (ARBs), Hemoglobin, Anemia, Chronic kidney disease (CKD)

## Abstract

**Background:**

Angiotensin converting enzyme inhibitors (ACEIs) and angiotensin receptor blockers (ARBs) are widely used in the management of congestive heart failure (CHF), diabetes mellitus (DM) and hypertension (HTN). Use of these agents is reported to cause anemia.

**Methods:**

We examined the association between standard care use of ACEI or ARB and subsequent change in hemoglobin (Hgb) in a population of 701 adult primary care patients with DM, CHF and/or HTN. Data analysis was conducted to adjust for baseline differences between the treatment groups.

**Results:**

After adjusting for differences in covariates at baseline between the subjects who were prescribed ACEI (N = 519) and ARB (N = 182), as well as the associated odds of being prescribed ARB, the ACEIs were associated with lower mean Hgb [0.18 (0.02, 0.34) g/dL, p = 0.02] at follow up relative to ARBs. However, patients with CHF experienced an increase in Hgb while on treatment (0.42 g/dL), especially those treated with ACEIs (0.56 g/dL). Chronic kidney disease at baseline was not associated with a significant decrease in Hgb in either treatment group.

**Conclusions:**

Since ACEIs and ARBs are most frequently used in patients who are vulnerable to complications from anemia, such as patients with CHF, HTN and DM, these findings may be useful to clinicians in selecting medications and monitoring patients for the adverse effects of treatment.

## Background

Angiotensin converting enzyme inhibitors (ACEIs) and angiotensin receptor blockers (ARBs) are antihypertensive drugs that are now in wide use for indications in addition to the control of hypertension [1,2]. This wide use is largely due to their renoprotective and cardioprotective effects in patients with diabetes mellitus (DM) and congestive heart failure (CHF).

In current practice ACEI/ARB medications are used for multiple reasons, ranging from prevention of proteinuria and progression to renal failure in diabetics, and first-line treatment of hypertensive patients with concurrent CHF and DM, to slowing the progression of heart failure and improving survival in CHF patients. There is a complex relationship between DM, CHF, and hypertension (HTN), as worsening diabetic nephropathy and CHF can lead to renal failure, causing HTN, further complicating the primary disease processes. ACEI/ARB medications have several uses in this complex situation, but the main goal is the prevention of complications, most notably renal failure leading to end stage renal disease.

In the advanced stages of diseases like CHF and DM, many patients develop some level of anemia. This is not a benign finding. Anemia contributes to the worsening of heart failure [3-5] and renal function [6,7] and in many instances the treatment of anemia becomes part of the management of the patient’s overall condition [8]. For example, the “Anemia Correction in Diabetes” (ACORD) trial demonstrated that correction of anemia prevented an additional increase in left ventricular mass, and was associated with a significant improvement in quality of life [8].

A systemic review and meta-analysis of the effects of anemia in heart failure patients found anemia to be associated with increased mortality in both systolic and diastolic CHF and suggested that anemia could serve as a useful prognostic marker [3]. Prevalence of anemia in CHF is highly variable. Depending on the severity of heart failure and diagnostic criteria for anemia, prevalence can be as high as 50% in selected patient cohorts [3]. Lower Hgb levels are associated with greater functional impairment and poor exercise tolerance. Patients with incident anemia have the poorest survival, followed by those with prevalent anemia and no anemia [4]. Analysis of Valsartan in Heart Failure Trial (Val-HeFT) data also suggests that patients with larger decreases in Hgb are at higher risk of hospitalization, morbidity and mortality [9].

A prospective hospital based study concluded that anemia is a significant predictor of decline in glomerular filtration rate (GFR) [6]. Decreased Hgb also serves to identify type 2 diabetic patients who are at increased risk of progression to advanced renal disease [6,10]. Overall, lower GFR is associated with lower Hgb level [11].

The current study was conducted to observe and compare the effects of ACEIs and ARBs on Hgb levels in adults with CHF, DM and/or HTN.

## Methods

This was a retrospective case series based on the electronic health records (EHR) of adult patients served by Essentia Health East in Duluth, MN. Eligible patients were aged 40–70, had an Essentia Health East primary care provider and received care within Essentia Health East facilities between July 1, 2004 and September 30, 2009. This study was reviewed and approved by the Essentia Health Institutional Review Board.

### Study population

To be included in the study, eligible patients were required to have (1) been initially prescribed ACEI or ARB medications between January 1, 2005 and December 31, 2008, without a documented discontinuation for at least 6 months; (2) a diagnosis of DM, CHF, and/or HTN; (3) documentation of baseline Hgb level (12 months before to 10 days after initiation of ACEI or ARB medication) and Hgb level during the follow up period (3 to 12 months after initiation of medication); and (4) baseline GFR or data needed to compute GFR (12 months before to 30 days after initiation of medication). ACEI use has been found to be associated with a decrease in erythropoietin concentrations after as little as 28 days; [12] in this study follow up Hgb was assessed in the 3 to12 month window following the initiation of therapy. There were 7042 eligible patients who were between 40 and 70 years of age at the time of treatment initiation.

Patients were excluded from the study if their medical record included evidence of (1) underlying conditions associated with anemia (hemoglobinopathy, bleeding disorder, chronic inflammatory disease, or treatment with vitamin B_12_ or folate) during the time from 6 months before initiation of ACEI or ARB until the end of the follow up period; or (2) other conditions or treatments that might affect Hgb level during the follow up period (blood transfusion, pregnancy, hemodialysis, malignancy requiring chemotherapy or radiotherapy, hospitalization, or treatment with EPO).

Following these exclusions, 5613 patients remained eligible for the study. Of these, 5104 met inclusion criteria (2), above, and 839 met both (2) and (3). The study population consists of the 701 patients who met all four inclusion criteria.

For eligible subjects data was extracted from the EHR: age, sex, class of medication (ACEI or ARB), underlying diagnoses (DM, CHF, and/or HTN), baseline Hgb and GFR, and follow up Hgb. If more than one Hgb report was recorded during the follow up period (3 to 12 months after the initiation of medication), the measure closest to 3 months was used. Chronic kidney disease (CKD) was defined as GFR < 60 mL/min/1.73 m [2].

### Data analysis

Preliminary analysis of the ACEI and ARB groups at baseline identified differences between the treatment groups with regard to sex; prevalence of HTN, CHF, and CKD; and baseline Hgb; although not all differences were significant at the 0.05 level. In addition, it was noted that both the rate of prescription of ARB vs. ACEI and the follow up Hgb decreased slightly over the four year period. Accordingly, the fractional number of years from January 1, 2005 until treatment was initiated was included as a variable in the analysis. The investigators recognized that in clinical practice, provider-selected treatment is rarely, if ever, random. The preferential ordering of ACEI or ARB for subpopulations defined by sex and co-morbidities was not due to specific treatment guidelines or standards of clinical practice. Accordingly, the treatment effect of ARB relative to ACEI was considered to be truly confounded by the covariates for which treatment group differences had been identified. It was thus necessary to consider the associations between the baseline covariates and both follow up Hgb and providers’ treatment choices, in order to produce an unbiased estimate of the treatment effect.

We estimated the effect of the two treatments on follow up Hgb with the use of the doubly robust (DR) semiparametric efficient estimator [13,14]. This method produces an estimate of the causal effect of ARB vs. ACEI on follow up Hgb by simultaneously incorporating (1) the chance that a given subject would receive ACEI or ARB, given their baseline characteristics (the propensity score), and (2) the effects of baseline characteristics (covariates) upon the outcome of interest (follow up Hgb). This approach to data analysis is “doubly robust” in the sense that the model produces an unbiased estimate of the causal effect if either (1) the effect of baseline covariates on the outcome or (2) the propensity score is correct. A complete-case ANCOVA was conducted. All analyses were completed using SAS ver. 9.2 and the SAS macro, %drmacro, described by Funk et al. [15] was used to produce the estimated causal effect and asymptotic confidence interval (CI). The bias-corrected and accelerated (BCa) bootstrap CI [16] of the causal effect was constructed for comparison with the asymptotic CI.

## Results

In all, EHR data for 701 patients met study inclusion and exclusion criteria, with 519 patients receiving ACEI treatment and 182 receiving ARB. On average, the two Hgb tests were 314 days apart and the second one was 227 days after the initiation of treatment. The mean age for all treated patients was 57.53 +/− 7.73, with more men (54.4%) than women in the study population. Table 1 provides a summary of the baseline demographic and clinical characteristics of the two treatment groups, with associated p-values.

**Table 1:** Demographics by study group

**Variable**	**Overall**	**ACEI group**	**ARB group**	***p*-*value***
	**(N = 701)**	**(N = 519)**	**(N = 182)**	
Age	57.53 +/− 7.73	57.45 +/− 7.74	57.75 +/− 7.72	0.6484
Sex (female)	320 (45.6)	221 (42.6)	99 (54.4)	0.0073
Hgb	14.58 +/− 1.43	14.67 +/− 1.39	14.32 +/− 1.51	0.0063
GFR	76.61 +/− 19.66	76.87 +/− 18.65	75.87 +/− 22.35	0.5898
CKD	118 (16.8)	80 (15.4)	38 (20.9)	0.1066
Diabetes mellitus	263 (37.5)	191 (36.8)	72 (39.6)	0.5339
Hypertension	657 (93.7)	481 (92.7)	176 (96.7)	0.0738
CHF	47 (6.7)	29 (5.6)	18 (9.9)	0.0572
Days between Hgb tests	313.68 +/− 120.77	312.34 +/− 118.40	317.50 +/− 127.56	0.6332
FUP days	226.70 +/− 81.39	226.02 +/− 82.34	228.64 +/− 78.83	0.7026

The estimated effects of baseline demographic and clinical characteristics on follow up hemoglobin are summarized in Table 2, both for the entire population studied (N = 701), and for the ACEI (N = 519) and ARB (N = 182) populations separately. Table 2 also summarizes the odds (odds ratio) that subjects with these characteristics received ARB (relative to ACEIs) treatment. The doubly robust analysis was based on these data.

**Table 2:** Impact of baseline covariates on (1) odds of receiving ARB relative to ACEI and (2) follow up Hgb levels (Factors Used in Doubly Robust Analysis)

		**Effect on follow up Hgb in g/dL (95% CI)**		
		**Overall**	**ACEI**	**ARB**
**Baseline covariate**	**OR (95% CI) for receiving ARB relative to ACEI**	**(N = 701)**	**(N = 519)**	**(N = 182)**
AGE	1.00 (0.98, 1.02)	0.00 (−0.01, 0.01)	−0.00 (−0.01, 0.01)	−0.00 (−0.02, 0.02)
Sex (female)	1.40 (0.96, 2.05)	−0.32 (−0.47, -0.17)	−0.29 (−0.47, -0.11)	−0.47 (−0.75, -0.20)
Diabetes mellitus	1.16 (0.81, 1.67)	0.01 (−0.14, 0.15)	−0.03 (−0.20, 0.15)	0.15 (−0.13, 0.42)
Hypertension	2.82 (1.12, 7.13)	0.29 (−0.01, 0.58)	0.21 (−0.12, 0.54)	0.49 (−0.24, 1.22)
CHF	1.90 (0.98, 3.68)	0.42 (0.14, 0.70)	0.56 (0.20, 0.91)	0.13 (−0.33, 0.58)
Hgb (pre)	0.89 (0.78, 1.02)	0.59 ( 0.53, 0.64)	0.60 (0.54, 0.66)	0.57 (0.48, 0.66)
CKD	1.19 (0.75, 1.89)	−0.15 (−0.34, 0.04)	−0.09 (−0.31, 0.14)	−0.31 (−0.64, 0.03)
Year of initiation of treatment	0.86 (0.74, 1.01)	−0.05 (−0.11, 0.01)	−0.05 (−0.13, 0.02)	−0.03 (−0.15, 0.09)

Sex, CHF, and baseline Hgb were associated with statistically significant effects on follow up Hgb. Patients with CHF were noted to have an increase in Hgb while on treatment (0.42 g/dl); this was especially noted in those treated with ACEIs (0.56 g/dl). While we observed that baseline CKD was associated with a decrease in Hgb (overall −0.15 g/dl, ACEI group −0.09 g/dl, ARB −0.31 g/dl), the difference between the two treatment groups was not statistically significant. In addition, ACEIs and ARBs were prescribed at different rates for different subpopulations, with ARBs prescribed more often for females and for subjects with an earlier treatment initiation date, as well as those with HTN, CHF, and with a lower baseline Hgb. Accordingly, (1) the likelihood that a subject with a specific set of baseline characteristics would receive ARBs (the propensity score), and (2) the observed follow up Hgb associated with each set of baseline characteristics were used in the doubly robust estimate of the effect of treatment on follow up Hgb.

The unadjusted mean Hgb for patients who had received ACEIs was 0.30 (0.21, 0.39) g/dL lower at follow up than at baseline (14.37 vs. 14.67 g/dL), while the unadjusted mean Hgb for patients who had received ARBs was essentially unchanged from baseline at 14.32 g/dL. Thus the raw (unadjusted) difference between the ACEI and ARB effects on Hgb was significant (p = 0.0008). After accounting for the effect of baseline covariates and adjusting for differential prescription of ACEI or ARB (propensity), the estimated follow up Hgb and 95% confidence intervals for ACEI and ARB treatment were 14.32 (14.21, 14.42) g/dL and 14.50 (14.35, 14.65) g/dL, respectively (see Table 3). The ACEIs were associated with lower mean Hgb [0.18 (0.02, 0.34) g/dL, p = 0.02] at follow up relative to ARBs. These results are consistent with those obtained by bootstrap, with 100,000 replications, which found an estimated causal effect of 0.18 (0.01, 0.33) g/dL. Chronic kidney disease at baseline was not associated with a greater decrease in Hgb in the study population as a whole, or in either treatment group.

**Table 3:** Follow up hemoglobin after ACEI or ARB treatment, adjusted for baseline covariates

**Treatment**	**Estimated follow up Hgb (g/dL)**	**Confidence interval**	***p*-*value***
ACEI	14.32	(14.21, 14.42)	
ARB	14.50	(14.35, 14.65)	
Difference	0.18	(0.02, 0.34)	0.02

## Discussion

We found that the use of ACEIs was associated with a decrease in Hgb levels, while the use of ARBs was not. After adjustment for the differences between the ACEI and ARB populations at baseline, the difference in follow up Hgb between the ACEI and ARB groups was found to be statistically significant. Since these drugs are most frequently used in patients who are vulnerable to complications from anemia, such as older patients with cardiac conditions, these findings may be useful to clinicians in selecting medications and monitoring patients for the adverse effects of treatment.

The mechanism of action of ACEIs and ARBs and their effects on hemoglobin level are not well established. Several studies have concluded that ACEIs may interfere with the production of erythropoietin (EPO) [12,17]. A study of kidney transplant patients concluded that ACEI related reduction in hemoglobin could reflect the modulation of multiple factors interacting with erythroid marrow progenitors [17]. An association between ACEI therapy, decrease in Hgb and suppression of EPO production has also been documented [18]. Angiotensin 2 increases proliferation of early erythroid progenitors but not the progenitors of other cell lines and ARB completely abolishes this effect [19]. These observations point to a possible inhibitory effect of these medications on bone marrow.

This study presented a challenge that is common in observational research: adjusting for baseline differences in treatment groups. That is, in determining the effect of ACEI and ARB medications on Hgb levels, we first had to adjust for the impact of other known factors. For example, both baseline Hgb and patient sex had larger effects on follow up Hgb than either of the classes of medications studied. Similarly, while the lower mean Hgb value at baseline in the ARB group (14.32 +/− 1.51) when compared to the ACEI group (14.67 +/− 1.39) might have been partially explained by the greater proportion of females in that group (54.4% vs. 42.6%), the magnitude of this effect was unknown. We also observed in this study that patients with CHF experienced an increase in Hgb while on treatment. This may be related to improvement in hemodilution in these patients as their CHF improves with ACEI therapy, as hemodilution is one of the possible etiologies of anemia in chronic CHF patients [20,21]. The reasons for the observed differences in the other covariates at baseline were also unclear, but ultimately could be rendered moot if the data analysis could fully adjust for the differences. The adjustment for baseline differences in covariates was the principal consideration in the selection of the analysis methods used in this study.

In planning this study, we were interested in determining if baseline level of renal function would affect the magnitude of the drop in Hgb with either of the classes of drugs studied. Worsening chronic kidney disease (CKD) is frequently associated with declining Hgb levels, and several studies have found that ACEI or ARB drugs may contribute to or exacerbate anemia [4,7,18,22]. In the current study, the presence of baseline CKD was found to be associated with a decline in Hgb in both ACEI (−0.09 g/dL) and ARB (−0.31 g/dL) patients, but the effect of CKD was not statistically significant. In 2008, Inoue et al. [7] reported on a retrospective chart review of Japanese patients using ACEI/ARB and found that the use of ARB was associated with a decrease in Hgb in patients with diabetic nephropathy. Of note is the study selection of patients with GFR < 60 mL/min/1.73 m [2]. As well-documented in the literature and National Kidney Foundation guidelines, advanced renal disease is associated with anemia independent of other risk factors. Therefore, we determined that it would be reasonable to study the effects of ACEI and ARB in heart failure and diabetic patients with early disease who have relatively well preserved kidney function.

Our finding that the use of ACEIs, compared with ARBs, was associated with a lower follow up Hgb after adjusting for covariates appears to be at variance with the findings of Inoue et al. [7] who reported a significant decrease in Hgb with ARBs (−0.54 ± 1.02 g/dL, p < 0.001), but not with ACEIs in their study of Type II diabetic patients with chronic kidney disease [7].

## Conclusions

We found that the use ACEIs was associated with a lower Hgb at follow up, while the use of ARBs was not. The difference was small but statistically significant. Clinicians should consider possible effects of ACEI and ARB therapy on Hgb, especially in ambulatory patients with unexplained anemia. An improved understanding of the association between the use of ACEI and ARB therapy and the development of anemia could contribute significantly to the management of patients with DM, CHF and HTN.

### Study limitations

This study was subject to several limitations. The sample (N = 701) was not large enough for extensive analysis of the effects of ACEI/ARB therapy in subgroups. In addition, while the study was designed to examine the impact of incident (new) use of ACEI and ARB medications, the study population may have included some individuals who were already receiving ACEI or ARB medications at baseline. The study population was limited to patients who already had primary care physicians at Essentia Health East prior to the initiation of ACEI/ARB therapy, so that the EHR could be used to exclude patients with known prior use of ACEIs/ARBs. This study was conducted in the Essentia Health East service area of Northwestern Wisconsin and Northeastern Minnesota, and the subjects doubtlessly reflected the limited ethnic diversity of the region.

### Ethical approval

This research was approved by the Essentia Health Institutional Review Board, Duluth, MN.

## Competing interests

The authors declare that they have no competing interests.

## Authors’ contributions

AA conceived of study, contributed to the study design, and drafted the manuscript. CEG contributed to the study design and drafted the manuscript. BPJ amended the statistical analysis plan, performed the statistical analyzes, and drafted the manuscript. CMR contributed to the study design, developed the statistical analysis plan, and drafted the manuscript. JAP procured and prepared the data. All authors read and approved the final manuscript.
